# Genotyping of *Trypanosoma cruzi* in a hyper-endemic area of Colombia reveals an overlap among domestic and sylvatic cycles of Chagas disease

**DOI:** 10.1186/1756-3305-7-108

**Published:** 2014-03-21

**Authors:** Ana María Mejía-Jaramillo, Luz Adriana Agudelo-Uribe, Juan Carlos Dib, Sylvia Ortiz, Aldo Solari, Omar Triana-Chávez

**Affiliations:** 1 Grupo BCEI, Universidad de Antioquia UdeA, Calle 70 No. 52-21 Medellin, Colombia; 2 ICBM, Facultad de Medicina, Universidad de Chile, Santiago de Chile, Chile

**Keywords:** Colombian indigenous communities, Risk factors for Chagas disease, Genotyping *Trypanosoma cruzi*, Seroprevalence, Sierra Nevada de Santa Marta

## Abstract

**Background:**

Chagas disease is a neglected illness caused by the *Trypanosoma cruzi* parasite, which widely affects American communities. This study attempted to identify *T. cruzi* genotypes circulating in four indigenous communities of the Sierra Nevada of Santa Marta, Colombia, to investigate parasite transmission dynamics in these communities. In addition, some epidemiological variables to determine the risk factors for infection with this parasite, such as the prevalence of *T. cruzi* infection, the triatomine species, and the domestic and sylvatic mammals that act as vectors and reservoirs of the parasite in the domestic, peridomestic and sylvatic cycles, were examined.

**Methods:**

We developed a prospective study to identify the main risk factors associated with *T. cruzi* infection in the region. The *T. cruzi* prevalence was determined by ELISA, IFA and PCR. Triatomines species and both domestic and sylvatic mammals from all communities were captured and sampled. To analyze parasite transmission dynamics in these four communities, eight DNA parasite probes were generated from insect and reservoir samples, and a DNA blot analysis were carried out.

**Results:**

Serological studies revealed 37% prevalence in the four communities, and Kasakumake was the most endemic region, containing approximately 70% seropositives. Moreover, the molecular diagnosis showed a high correlation between the serological data and the *T. cruzi* circulating in the patients’ blood. A total of 464 triatomine insects were collected in domestic, peridomestic and sylvatic environments, and these insects belonged to five different species; *Rhodnius prolixus* and *Triatoma dimidiata* were the two more important species transmitting the parasite. After studying the eco-epidemiological factors in these four communities, the most important risk factors for infection with the parasite were determined. These risk factors are a high infection rate of people and domestic animals, the construction materials of the houses, the presence of infected triatomines inside the human dwellings, the proximity between houses and a sylvatic environment with several triatomine species and wild animals. Finally, the molecular characterization of *T. cruzi* showed the presence of three haplotypes and complex *T. cruzi* mixed infections in all reservoirs.

**Conclusions:**

Active transmission of *T. cruzi* is present in four indigenous communities of the Sierra Nevada de Santa Marta with overlap between the domestic and the sylvatic transmission cycles of Chagas disease.

## Background

Chagas disease, caused by *Trypanosoma cruzi*, is a complex systemic disease that affects more than 8 million people, resulting in approximately 10,000 deaths each year in Latin America [1]. Indigenous communities are particularly at risk for infection due to their social, ecological, environmental and cultural conditions, which are advantageous for the establishment of the insect vectors and the possible transmission of the parasites [2-5].

The Sierra Nevada de Santa Marta (SNSM) is located in the northeastern region of Colombia between a latitude of 10 and 11 degrees north and a longitude of 72 and 74 degrees west and an altitude between 200 and 1,500 m. The SNSM has one of the most special and unique geographies in the world because it contains the entire thermal zone and all six biomes of Colombia. It has humid forests with hot, temperate, cold thermal zones as well as paramo, superparamo and nival plains, which engender a wide diversity of floral and faunal species and nurture various endemic species.

Several comprehensive works have suggested that the SNSM involves complex eco-epidemiological scenarios which show different *T. cruzi* prevalence rates in humans among the indigenous communities [4,6], divergent intradomiciliary-found triatomine species that feed on the human dwellers [3,4,6-8] and distinct *T. cruzi* genotypes circulating between the domestic and sylvatic transmission cycles [9,10].

Although these studies reported active transmission of *T. cruzi*, no large surveys have been conducted to understand the epidemiology of Chagas disease in this zone. To this end, we extended the previous studies of four indigenous communities of SNSM (Manzanal, Kasakumake, Gumake and Umandita) [10]. For this study, we analyzed parasite transmission dynamics in these communities using eight DNA parasite probes generated from insect and reservoir samples to identify the circulating *T. cruzi* genotypes. Additionally, the main risk factors and prevalence of *T. cruzi* infection were determined and the triatomine species and both the domestic and sylvatic mammals that act as vectors and reservoirs of the parasite in the domestic, peridomestic and sylvatic cycles were identified. Herein, we present new evidence of the overlap between the domestic and sylvatic transmission cycles in this hyperendemic region of Colombia. These parameters inform the implementation of appropriate control measures in this zone to combat the basic health problems of these communities.

## Methods

We developed a prospective study to identify the main risk factors associated with *T. cruzi* infection. This study was carried out over two years with two surveys per year. During visits to the study area, numerous activities were conducted, including human serological and epidemiological studies, animal surveys, sylvatic and domestic animal captures, and triatomine collections.

### Study sites and human populations

The study was conducted in the SNSM, located on the basin of the Palomino River at an altitude between 200 and 1500 m (10°01’05”-11°20’11”N and 72°36’16”-74°12’49”W), where the Manzanal, Kasakumake, Umandita and Gumake communities are located [10]. All four villages have a certain degree of deforestation, and the presence of *Attalea butyracea* palms is common to all four. Additionally, there are other conditions that facilitate the presence of triatomine bugs, such as rock formations, a great diversity of wild and domestic animals and the construction materials of local dwellings. All houses are built on uncoated floors with walls made of cane (*Gynerium sagittatum*), mud or wood and woven roofs made from palm leaves. The population consists of three indigenous ethnic groups, the Kogis, the Wiwas and the Arhuacos. The first group inhabits Manzanal and Umandita, and the second and third groups live in Kasakumake and Gumake, respectively. All individuals aged 1–100 years living in these communities were invited to participate in this study.

### Ethical approval and informed consent

The protocol was approved by the Human Ethics Committee of the University of Antioquia, Colombia. The objectives of this study were explained to the residents and all participants signed an informed consent form.

### Serosurvey data collection

The serological survey was conducted to obtain information on *T. cruzi* prevalence in the populations studied. The age, sex, and place of residence were recorded for each participant. A 5-ml blood sample (3 ml for children under 5 years) was drawn. The blood was separated by centrifugation, and the serum and the cells were stored at −20°C in separate aliquots. The sera were screened for antibodies to *T. cruzi* with an enzyme-linked immunoabsorbent assay (ELISA) and an indirect immunofluorescent assay (IFA) [11]. The specimens positive by both ELISA and IFA were considered infected with *T. cruzi*. The serological data were analyzed using Epi Info 6 Version 6.04a (CDC, Atlanta, USA).

### In-house ELISA

The cytosolic fraction of the *T. cruzi* epimastigotes from a mixture of the I.RHO/CO/00/CAS-15.CAS and I.TRI/CO/03/MG-8.MAG Colombian strains was used as the antigen. Briefly, Nunc microtiter plates were sensitized overnight at 4°C with 100 μl of the antigen solution in 0.05 M carbonate-bicarbonate buffer (pH 9.6) at a concentration of 200 ng/ml. The sera were diluted 1/100 in phosphate-buffered saline–Tween 20 (0.05%; PBST), and 100 μl of this mixture was added to the wells. After one hour at 37°C, the plates were washed three times with PBST, anti-human IgG-peroxidase conjugate (Sigma-Aldrich, Missouri, USA; 1/10,000 dilution in PBST) was added to the wells, and the wells were incubated at 37°C for an additional hour. After three additional washes, the immune complexes were developed with a citrate phosphate (pH 5.0), o-phenylenediamine (OPD; Sigma-Aldrich, Missouri, USA), and H_2_O_2_ buffer, and the absorbance was read at 450 nm. The cutoff values were calculated as the average absorbance of two negative controls plus two times the standard deviation, as was previously described by Guhl and Nicholls, 2001 [11].

### Indirect immunofluorescent assay (IFA)

The positive sera from the first test were analyzed using IFA following a previously described procedure [11]. The IFA was performed using the formaldehyde-treated epimastigote forms of *T. cruzi* (from a mixture of I.RHO/CO/00/CAS-15.CAS and I.TRI/CO/03/MG-8.MAG Colombian strains) as the antigen and a FITC anti-human IgG conjugate (Biomérieux, Bogotá, Colombia). The IFA was considered inconclusive when the titer was 20 and positive when it was at least 40. The specimens that gave an uninterpretable result at this dilution were considered inconclusive.

### Triatomine collections and *T. cruzi* infection

An entomological search to collect triatomines in domestic, peridomestic, and sylvatic areas was carried out. The specimens were collected by timed manual collections, light-trapping (Shannon) [12], Noireau traps [13], and collection by local householders. The timed manual collections consisted of searching for triatomine bugs inside homes and peridomestic areas. The intradomiciliary searches mainly focused on wall cracks, floors, roofs, and beds for 30 min in each house by two persons. The peridomestic structures were searched for 30 min by two persons and included corrals for pigs, chicken coops, trees where chickens roosted, storerooms, woodpiles, stones, wooden fences, kitchens, and other possible refuges for triatomines within the area of human activity. The light-trapping collections were performed outside the houses, and each of the surveys was performed for 2 hours per person per night. The Noireau traps were placed in some common areas, such as schools or meeting places, throughout the night for two weeks. Mice were used in the live-bait traps and they were checked daily [13]. The householders were encouraged to collect invading triatomines and were given plastic bags to store the insects until the next visit. The triatomine survey was conducted with the help of the inhabitants of each house. Finally, the sylvatic environment, mainly palm trees and rocks, were searched for insects by using live-bait traps [13].

All bugs were placed in plastic bottles, marked and identified by species and stage at the laboratory, according to the classification proposed by Lent and Wygodzinsky (1979) [14].

All live insects were individually examined for *T. cruzi* infection within 7 days of capture. For this examination, triatomine feces were taken by abdominal compression, diluted with a phosphate-buffered saline (PBS, pH 7.0) solution and microscopically examined.

### Identification of risk factors

During the triatomine sampling, a survey was conducted to identify the risk factors associated with triatomine presence in houses and the transmission of *T. cruzi*[15-17]. This was performed in 25 houses in the Gumake community because this community was easier to access. Data on the location of the house, distance from other houses, surrounding vegetation, animals on the property, previous entomological results and the roof, wall, and floor construction materials were collected. To identify the risk factors associated with the presence of triatomines in houses, a multiple regression analysis was carried out using the Statgraphics 4.0 program, which provides exact regression estimates, 95% CIs, and *p*-values <0.05.

### Domestic and sylvatic animal surveys

Domestic and sylvatic animals were processed according to the Institutional Animal Ethics Committee at the University of Antioquia, Colombia. The samples from the domestic animals were taken during visits to the farms of the indigenous areas only in Gumake and Umandita. A house-to-house census of all dogs and cats was undertaken in all houses. With the consent of their owners, the animals were bled and taxonomically classified [18]. The mammalian blood samples (0.2-5 ml of blood with 6 M guanidine hydrochlorideand 0.2 M EDTA) were used to diagnose *T. cruzi* infection by PCR as outlined below.

To capture the sylvatic mammals, National Sherman folding aluminum double-door traps (Tomahawk® and Sherman®) for sampling small and medium-sized animals were used in the Gumake community [19-21]. The traps were located in longitudinal transects in forests of different succession stages along roads and streams, starting from the peridomestic area of the houses to the entrance into the extradomestic area, or forest. There were a total of six transects with 7–17 traps placed in each. A total of 74 traps were placed, and the site of each trap was marked and coded on a sketch showing the location of the traps, which facilitated daily review. The animals were captured and taxonomically identified [18]. Their blood samples were analyzed for the presence of *T. cruzi* as described for domestic mammals. The wild animals were released after this process.

### PCR diagnosis and transmission dynamics analyses

The feces samples obtained directly from the insect vectors and the DNA from the patients blood samples and the domestic and sylvatic animals blood samples were used to detect *T. cruzi* infection by kDNA variable region PCR amplification. Additionally, the infected insects were used for parasite isolation through the inoculation of feces into mice. After the haemoculture the *T. cruzi* stocks were isolated in LIT culture medium.

### DNA extraction

DNA from the patient and reservoir blood samples and the *T. cruzi* strains from cultures was extracted using the phenol-chloroform (Amresco, Solon, Ohio, USA) method and precipitated with ethanol [22]. The DNA from 25 μl of triatomine feces was purified using the DNAzol reagent (Gibco BRL, Gaithersburg, MD), as recommended by the manufacturer.

### Amplification of the hypervariable region of the minicircles

Amplification was performed in 0.2-ml microcentrifuge tubes containing 25 μl of the reaction mixture. Previously described 121 (5′-AAATAATGTACGGGT/GGAGATGCATGA-3′) and 122 (5′-GTTCGATTGGGGTTGGTGTAATATA-3′) primers were used for the amplification of the variable region of the minicircles from different biological samples [23]. These primers allow amplification of a 330-bp fragment. The reaction mixture contained 1 μl of DNA template, 1× Taq buffer, 1.5 mM MgCl_2_, 200 μM dNTPs, 0.2 μM of each primer, and 1 U of *Taq* polymerase (Fermentas, Ontario, Canada). The amplifications were performed on a PTC-100 programmable thermal cycler (MJ Research Inc., Waltham, MA) programmed for an initial denaturation step at 94°C for 3 min, followed by 35 cycles of 94°C for 45 s, 63°C for 45 s and 72°C for 45 s, and a final cycle at 72°C for 10 min. The amplified products were analyzed by electrophoresis on a 1% agarose (Amresco, Ohio, USA) gel stained with ethidium bromide, visualized under UV light, transferred onto nylon membranes, and cross-linked by UV irradiation.

### Molecular typing by mini-exon genes

To identify *T. cruzi I* (TcI) or other Discrete Typing Units (DTUs) in the positive samples, a multiplex PCR on an intergenic spacer from a mini-exon gene was performed. The primers for these amplification were: 5′-GTG TCC GCC ACC TCC TTC GGG CC-3′ (TC1, groups II-VI-specific), 5′-CCT GCA GGC ACA CGT GTG TGT G-3′ (TC2, group I-specific) and 5′–CCC CCC TCC CAG GCC ACA CTG-3′ (TC, common to both group I and group II-VI) [24]. Amplicons of 350 bp for TcI and 300 bp for TcII, TcV or TcVI were expected [25]. The PCR was performed in a final reaction volume of 25 μl, containing 1× Taq buffer, 1.5 mM MgCl_2_, 200 μM of each dNTP, 0.4 μM of each primer, 0.625 U of *Taq* polymerase (Fermentas, Ontario, Canada) and 2.5 μl of DNA. The PCR was carried out at an initial temperature of 94°C for 1 min, followed by 26 cycles of 94°C for 30 s, 55°C for 30 s, and 72°C for 30 s, with a final extension at 72°C for 10 min [24]. The amplified products were analyzed as above.

### Identification of TcI Haplotypes

To identify the TcI circulating haplotypes in the study area, we amplified the mini-exon region with primers 1-A (5′-TGTGTGTGTATGTATGTGTGTGTG-3′) and 1-B (5′-CGGAGCGGTGTGTGCAG-3′); 2-A (5′-TGTGTGTGTGTATGTATGTATGCT-3′) and 2-B (5′-GGAACACACGCGACTAAT-3′); and 4-A (5′-CTGCAGGCACACGTGTGT-3′) and 4-B (5′-AAAAGACGGGAAAAAAGCAA-3′) to distinguish between haplotypes Ia, Ib and Id, which amplify 228 bp, 250 bp and 200 bp products, respectively, as described previously [26].

### DNA blot analysis

DNA blot analysis was used with 178 *T. cruzi* DNA samples to determine the transmission dynamics of the parasite genotypes. Eight selected DNA probes from the samples with a wide diversity of different LSSP-PCR profiles were selected [10]. The probes were obtained by amplification of the minicircle variable region of *T. cruzi* isolated from different samples: V19 and V25 probes of *R. prolixus* from Umandita and Gumake, respectively; V53 and V5 of *T. dimidiata*, and R30 and R62 of *Canis lupus familiaris* and *Heteromys anomalus*, respectively, all from Gumake; and G53 and K24 of humans from Gumake and Kasakumake, respectively. The primers for probe generation were CV1 (5′-GATTGGGGTTGGAGTACTAT-3′) and CV2 (5′-TTGAACGGCCCTCCGAAAAC-3′), which produced a 270-bp fragment. These fragments were further digested with restriction endonucleases Sau96I (Fermentas, Ontario, Canada) and ScaI (Fermentas, Ontario, Canada) to obtain a 250-bp band and remove all sequences from the minicircle constant region. Finally, the probes were labeled using the random primer method with [α-^32^P] dCTP. The membranes were prehybridized in a solution of 6× SSC and 1 mM EDTA, pH 7.4, with salmon-sperm DNA at 55°C. The membranes were hybridized overnight with the labeled probe at 55°C in the same solution and then washed in high-stringency conditions (0.1× SSC and 0.1× SDS at 68°C). The membranes were exposed for 1–4 h in the Molecular Imager (FX Bio Rad Laboratories, Hercules, CA). This method has been validated by hybridization with the probes constructed by PCR amplification of *T. cruzi* DNA from different DTUs [10,27-29]. The hybridization profiles were analyzed to compare the intensity of the ethidium bromide-stained bands in each membrane with the presence of the radioactive bands obtained with each probe. The specificity of the probes was tested in hybridization controls against DNA of the probes itself. The profiles of radioactive hybridization signals with multiple probes are indicative of mixed infections with several lineages of *T. cruzi.*

## Results

### Diagnosis of *T. cruzi* infection in indigenous groups

The serum samples of 214 individuals from the four communities, which represent 34% of the total population, were analyzed by ELISA and IFA. The seroprevalence values observed were 22.7%, 77.8%, 26.8% and 36.2% for Manzanal, Kasakumake, Umandita and Gumake, respectively (Table 1), with a global prevalence of 37% in the area. There were no statistically significant differences between men and women (*p* > 0.05) in the four communities and between people younger than 15 years old and over 16 years old (*p* > 0.05) in the first three communities; however, in Gumake (*p* = 0.00005), the prevalence was higher in the older age group. The seroprevalence in the different indigenous ethnic groups was highest for the Wiwas (77.8%) followed by the Arhuacos (36.2%) and finally the Kogis (25.8%).

**Table 1:** Seroprevalence in the indigenous people from the four communities of SNSM, Colombia

**Community**	**Total population**	**Population tested**	**Prevalence IgG ELISA-IFI**
Manzanal (Kogis)	90	22/90 (24.4%)	5/22 (22.7%)
Kasakumake (Wiwas)	150	27/150 (18%)	21/27 (77.8%)
Umandita (Kogis)	90	71/90 (78.9%)	19/71 (26.8%)
Gumake (Arhuacos)	300	94/300 (31.3%)	34/94 (36.2%)
**TOTAL**	**630**	**214/630 (34%)**	**79/214 (37%)**

Moreover, 99% of individuals (204 of 214) were additionally diagnosed by PCR using kDNA. In this case, the rates of positive results were 22.7%, 55%, 21% and 46% for Manzanal, Kasakumake, Umandita and Gumake, respectively, with a concordance of 90.5% between the serological and parasitological PCR diagnoses.

### Entomological studies and diagnosis of *T. cruzi* infection

A total of 464 triatomine specimens were collected in domestic, peridomestic, and sylvatic areas, corresponding to five species. In Umandita, *R. prolixus*, *Rhodnius pictipes*, and *T. dimidiata* were collected, and in Manzanal and Kasakumake, only *R. prolixus* was found. The *R. prolixus* species was predominant in the domestic environments in the four communities, but in Gumake, *T. dimidiata* was also widely found. *R. pictipes* was a new species found in the study area, and *Panstrongylus rufotuberculatus* was found in the peridomestic areas. One of the four rock formations showed *Panstrongylus geniculatus*, and *T. dimidiata* specimens were collected from four of the 12 dissected *A. butyracea* palm trees (Table 2). In Gumake, all five species were collected. The percentages of *R. prolixus* nymphs found within the total number of insects captured within the human dwellings of Manzanal, Kasakumake, Umandita and Gumake were 73%, 56%, 47% and 52%, respectively (Table 2). The infection indices of the insects collected in all the areas were 13.6% (Manzanal), 8.8% (Kasakumake) 7.98% (Umandita) and 18.3% (Gumake) (Table 2).

**Table 2:** Entomological triatomine survey from the four communities of SNSM, Colombia

**Community**	***R. prolixus***				***R. pictipes***		***T. dimidata***				***P. geniculatus***		***P. rufotuberculatus***
	**D**		**P**	**Tc**	**D**	**P**	**D**	**P**	**S**	**Tc**	**D**	**S**	**P**
	**AD**	**N**	**AD**	+	**AD**	**AD**	**AD**	**AD**	**AD**	+	**AD**	**AD**	**AD**
Manzanal	12	32	0	6	0	0	0	0	0		0	0	0
Kasakumake	65	83	5	13	0	0	0	0	0	0	0	0	0
Gumake	20	22	0	9	6	2	32	7	10	10	1	2	**2**
Umandita	82	72	0	13	0	2	1	6	0		0	0	0
**TOTAL**	**179**	**209**	**5**	**34**	**6**	**4**	**33**	**13**	**10**	**10**	**1**	**2**	**2**

D: Domestic

P: Peridomestic

S: Sylvatic

Tc +: Positive for *T. cruzi*

AD: Adults

N: Nymphs

Finally, the transmission risk indices, which were estimated in 83.3% of the total dwellings from the four communities, were calculated. The dispersion index was 100% in the entire region studied, and the domiciliary infestation, peridomiciliary infestation, and colonization indices were 24.6%, 18.6% and 87.5%, respectively. Finally, the density and dwelling indices were 5.49 and 26.75, respectively (Table 3).

**Table 3:** Risk factor studies on the four communities of SNSM, Colombia

**Community**	**Dwellings analyzed**	**Domiciliary infestation index**	**Peridomiciliary infestation index**	**Colonization index**	**Density index**	**Crowding index**
Manzanal	6/6 (100%)	2/6 (33.33%)	nd	2/2 (100%)	44/6 (7.33)	44/2 (22)
Kasakumake	7/7 (100%)	3/7 (42.86%)	2/7 (28.57%)	3/3 (100%)	148/7 (21.14)	148/3 (49.3)
Gumake	25/38 (65.8%)	8/25 (32%)	8/25 (16%)	6/8 (75%)	81/38 (2.13)	81/8 (10.13)
Umandita	27/27 (100%)	3/27 (11.11%)	1/27 (3.70%)	3/3 (100%)	155/27 (5.74)	155/3 (51.6)
**TOTAL**	**65/78 (83.3%)**	**16/65 (24.6%)**	**11/59 (18.6)**	**14/16 (87.5%)**	**428/78 (5.49)**	**428/16 (26.75)**

### Studies of risk factors associated with domestic and peridomestic features

In 16 of the 25 houses surveyed from Gumake community, there were people with positive serology for *T. cruzi*, and in two of the houses, triatomines infected with the parasite were found. Twenty-one houses had thatched roofs, 17 had wooden walls, and 18 were 20–500 m from other dwellings. With these data, multiple regression and principal component analyses were performed to identify potential risk factors associated with the presence of triatomines and the transmission of *T. cruzi*. The logistic regression analysis showed a positive association between seropositive status with age (*p* < 0.05) and the roof construction material (*p* < 0.05). On the other hand, principal component analysis indicated that infected triatomines, roof material, and distance between houses were the most important variables contributing to the risk of infection with *T. cruzi*, with values of 53.8%, 48.4%, and 43.15%, respectively.

### Diagnosis of *T. cruzi* infection in animals

A total of 135 domestic animals were sampled, primarily from Gumake (117) with a few from Umandita (18). The species studied included *Canis lupus familiaris* (108 from Gumake and 18 from Umandita) with *T. cruzi* infection prevalences by kDNA PCR of 50% for both communities. *Felis catus* (5) had a 40% prevalence of positive results while there was one *Sus scrofa* (1) and no *Cavia porcellus* (3) infections. Five of the positive dogs showed signs of the disease (hair loss, ulcers, nail overgrowth, nasal depigmentation, weight loss, and inactivity). The dogs were active in the fields and hunting with their owners. It is important to emphasize these data because triatomines were not found in the resting places of these animals. However, 12 dogs, one pig, and one cat belonged to households where some of the inhabitants were seropositive. In addition, 16 sylvatic mammals from Gumake were surveyed: 14 rodents and two marsupials. They belong to the species *Proechymis semiespinosus* (6)*, Dasyprocta punctata* (1)*, Heteromys anomalus* (7)*,* and *Didelphys marsupialis* (2), and 50%, 0%, 43% and 50%, respectively, were positive for *T. cruzi* infection by kDNA PCR.

### *Trypanosoma cruzi* genotyping and transmission dynamics

**Figure 1:**
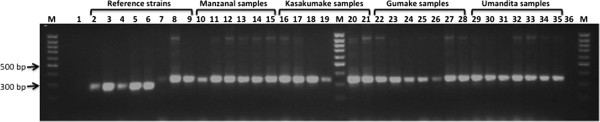
**Genetic characterization of the parasites by the amplification of the intergenic regions of the mini-exon genes.** Agarose gel electrophoretic analysis of the amplification products from the reference strains belonging to TcII (2–3), TcIV (4–5), TcVI (6), TcI (7–9) and different samples isolated from the Manzanal (10–15), Kasakumake (16–21), Gumake (22–28) and Umandita (29–35) communities of SNSM, Colombia. Samples 1 and 36 are negative controls, and M is the 100-bp ladder molecular weight marker.

To understand more about the *T. cruzi* transmission dynamics in this area and to propose appropriate control strategies, a molecular study was designed. First, the kDNA positive samples obtained from patients, triatomine feces, and domestic and sylvatic animal blood as well as the *T. cruzi* stocks isolated from infected triatomines were characterized by mini-exon amplification. All biological samples in addition to the ten isolated *T. cruzi* stocks (two from *T. dimidiata* of Gumake, two from *R. prolixus* of Umandita, four from *R. prolixus* of Kasakumake, and two from *R. prolixus* of Manzanal) were classified as *T. cruzi* I with this marker (Figure 1). Interestingly, we found one of the *T. cruzi* stocks isolated from sylvatic *T. dimidiata* belongs to the TcIb haplotype and one isolated from *R. prolixus* of Manzanal was TcId, while the other three isolated from the same vector of Umandita and Kasakumake were classified as TcIa (data not shown).

To study *Trypanosoma cruzi* transmission dynamics, we used eight kDNA positive biological samples selected from infected humans, vectors and reservoirs, and prepared radiolabeled DNA probes to be used in hybridization tests with PCR-DNA blotting from 178 infected hosts of the SNSM. The hybridization profiles obtained demonstrated that some of the probes discriminate between samples from different hosts in certain localities (Table 4). The probes V19 and V53, which were amplified from insect samples, reacted poorly with the human samples (0–23.4%), and reacted to a greater extent with vectors (15.38-50%) and reservoirs samples (21.87-77.77%) from all localities. The other probes from insects, V25 and V5, did not display any discriminatory power as they hybridized well with all samples. A different situation was observed with the probes obtained from animals. For instance, the R30 probe isolated from Gumake, reacted very poorly with the human samples from Gumake (8.51%) but rather well with the human samples from other localities (23.8-37.5%) as well with vectors (47.05-72.72%) and reservoirs (44.44-56.25%). Additionally, the R62 probe, showed no reaction with the human samples and the reservoirs of Umandita, reacted very poorly with the human samples of Kasakumake (9.52%), yet reacted better with samples of other localities (15.62-81.81%). The probe K24 obtained from human of Kasakumake hybridized very poorly with the reservoir samples from Gumake (6.25%) and highly with samples of all localities (22.22-90.47%). Finally, the other human probe, G53, reacted very poorly with all samples (0–11.11%). Interestingly, patient samples from Manzanal showed no reactivity with all the probes, except the V25.

**Table 4:** Hybridization results from the reactivity of eight probes with different samples from the four communities of SNSM, Colombia

**PROBES>**	**V19**	**V25**	**V53**	**V5**	**R30**	**R62**	**G53**	**K24**	
**SAMPLES**									**COMMUNITY**
Patient	23.40	53.19	4.25	34.04	8.51	34.04	6.38	27.65	GUMAKE
Vector	35.29	64.70	29.41	35.29	47.05	35.29	0	47.05	
Reservoir	28.12	31.25	21.87	34.37	56.25	15.62	3.12	6.25	
Patient	6.25	62.50	1.87	50.00	37.50	0	6.25	75.00	UMANDITA
Vector	38.46	30.76	15.38	38.46	69.23	46.15	0	46.12	
Reservoir	77.77	33.33	55.55	44.44	44.44	0	11.11	22.22	
Patient	0	50.00	0	0	0	0	0	0	MANZANAL
Vector	50.00	33.33	16.66	33.33	50.00	16.66	0	33.33	
Patient	14.28	47.61	9.52	52.38	23.80	9.52	4.76	90.47	KASAKUMAKE
Vector	27.27	18.18	36.36	54.54	72.72	81.81	0	36.36	

## Discussion

Chagas disease is one of the most important parasitoses in Latin America and a major cause of morbidity and death in affected countries [1]. Currently, it is important not only to know the *T. cruzi* seroprevalence in the different affected communities but also to understand the parasite transmission dynamics so that appropriate control strategies for the study area can be developed [30-34].

Previous screening studies conducted in other indigenous communities in the north of the SNSM showed a *T. cruzi* seroprevalence of 45.7% [4]. In the present study, we not only increased the number of studied people but also the number of communities and other parameters in addition to the epidemiological study, such as the sylvatic cycle, the genotyping of the parasite and the circulation of different *T. cruzi* genotypes between hosts and communities. We found a 37% seroprevalence in the four communities and conditions that favor the establishment of the vector and parasite transmission between different cycles. Kasakumake was the community with the highest prevalence (77.8%), which can be explained by the highest domiciliary, peridomiciliary and density vector indices of the four communities. Therefore, given the epidemiological data for Colombia regarding Chagas disease [35,36], the serological results obtained in this research are exceedingly important because they show a high level of *T. cruzi* transmission.

Another important result was found in the seroprevalence of the Kogis ethic group. Parra et al. (2004) showed no seroreactivity in this ethnic group, which is settled in the Gogtsezhi and Kemakumake communities [4]. However, our results show that although this group has the lowest *T. cruzi* prevalence (25.8%) of the three indigenous ethnicities, it is infected with the parasite. This result is very important because the members of this ethic group frequently visit members from other communities, making them more likely to become infected with the parasite and spread this infection between the people of their own communities.

Additionally, regarding the serological results, there were statistically significant differences between the two different age groups in the Gumake community. These results are consistent with those reported by Dib et al. (2006), which showed an increase in seroprevalence depending on age in other basins of the northern slopes of the SNSM [3]. In the social organization of the communities studied, the young people over 15 years old are considered as grown-ups. Consequently, they participate in a greater number of adult activities, such as hunting, forest raids, and evening meetings in the main house, which could be risk factors for the transmission of Chagas disease.

Although ELISA and IFA are the accepted techniques for the diagnosis of *T. cruzi* infection [11], it is not possible to detect circulating parasites in the blood using these methods. In the present study, we also used PCR as an approach to determine the prevalence of bloodborne parasites [37]. Given the high percentages of positive results obtained with this test, the parasites may be circulating in blood of these individuals. This could not only increase the risk of transmission to a non-infected individual but also the risk of infection with different strains of the parasite, which would maintain the activity of different transmission cycles.

With the entomological risk indicators measured, the dispersion index of 100% is notable. This index indicates an influx of insects within the basin of the Palomino River, which is a sign of the potential risk of transmission of *T. cruzi* among neighboring populations. This was also confirmed in a recent study of *T. cruzi* genotypes with a smaller sample size circulating in the insect vectors and human populations in the four communities [10]. Similarly, the rates of colonization found – 100% for Manzanal, Kasakumake and Umandita and 75% for Gumake – show that people are living with triatomines. Therefore, it is possible that a domestic transmission cycle is occurring due to the infection indices of greater than 8% found in this work.

The roof construction material of the houses in Gumake, most often made from palm leaves (*A. butyracea*), is an important risk factor for human *T. cruzi* infection because in this particular location, these palms were positive for *T. dimidiata*. Additionally, resident species (vertebrates and/or invertebrates) infected with *T. cruzi* were found in this type of roof in some of the households surveyed, showing that roofs are an important artificial refuge for triatomines [7,38,39]. The typical landscape of the domestic cycle of Chagas disease in Latin America is made of rural areas where the houses have roofs made of straw or palm; walls of mud, stone, or wood; and floors of dirt and mud and typically lodge a very poor human population. Human infection is linked to the sociocultural conditions of the people who are in close contact with the vector and the parasite [40,41]. Thus, after studying the eco-epidemiological factors of these four communities, the results suggest that there are many risk factors for parasite infection. These risk factors include (but are not limited to) a high infection rate of people and domestic animals, the construction materials of the houses, the presence of infected triatomines inside the houses, the proximity between houses and a sylvatic environment with several triatomine species and infected wild animals.

To extend our study, we used molecular tools, such as mini-exon amplification, to determine the TcI haplotypes circulating in the area, and *T. cruzi* genotyping, using hybridization tests with eight different DNA probes. Interestingly, the results obtained with the mini-exon marker showed the presence of the three TcI haplotypes commonly found in Colombia [26]. However, two of them (TcIb and TcId) were not associated with their transmission cycle as they have been previously described [26], suggesting the overlapping of transmission cycles. To broadly address this aspect, in the present work, we generated probes that varied from those with low selectivity, which recognized large numbers of samples, to those that did not discriminate the *T. cruzi* lineages circulating in different hosts and geographical areas (V25 and V5), to those that were very selective (G53). Other probes had partial selectivity, which allowed the discrimination of samples from some types of hosts and/or localities (V19, R30, R62 and K24). Interestingly, the two more promiscuous probes (V25 and V5) were generated from parasites isolated from *R. prolixus* and *T. dimidiata* and confirmed that these two vectors are responsible for transmitted *T. cruzi* in this zone. Surprisingly, almost all probes recognized the stocks isolated from vectors that belong to the three *T. cruzi* TcI haplotypes found in Colombia.

The hybridization results obtained with the more selective probes suggest that the variability of the *T. cruzi* lineages is not associated with the geographic locations considered but rather with the type of host. However, most of the probes hybridized simultaneously with samples from humans, vectors and reservoirs, indicating that most of the samples chosen for probe generation were mixed infections. One exception is the human G53 sample, which showed poor but very selective reactivity when used as a DNA probe in the hybridization tests. Therefore, the variability of the *T. cruzi* genotypes was higher in the invertebrate hosts than in the patients and the reservoirs. An interesting result occurs with the *T. cruzi* genotype circulating in patients of Manzanal. Very few human samples hybridized with the DNA probes except with V25. This result probably indicates the presence of another different *T. cruzi* genotype circulating in patients but not in vectors of that community. Manzanal is far from the other communities even though they belong to the same ethnic group of Umandita [10]. This variability agrees with the observation of *T. cruzi* genotype selection in vertebrate hosts when infected with multiclonal *T. cruzi* infections [42].

Taken together, the results obtained here, with a panel of eight probes chosen from different hosts in the SNSM, indicate a high level of overlap between the *T. cruzi* domestic and sylvatic transmission cycles as well as complex mixed infections. These complex *T. cruzi* mixtures are properly differentiated by minicircle hybridization, which was used as described in previous studies containing varied compositions of *T. cruzi* genotypes in other endemic areas [43,44]. In addition, a previous survey demonstrated that probes derived from the minicircle variable region of two cloned *T. cruzi* lineages hybridized only with the clones belonging to the same lineage [45]. This indicates that the resolving power of this method is high and it allows for the resolution of mixed infections. Moreover, it has been demonstrated in other geographic areas with this type of genotyping that in many cases, there is hybridization with more than one probe, suggesting that mixed infections are frequent in vertebrate and invertebrate hosts [46,47].

Finally, given the strong social structure as well as the political and cultural life of the communities, we propose the development of a prevention and control program for Chagas disease arising from the real needs of each community that does not clash with their beliefs and convictions. Such a program should be developed in conjunction with the health authorities of competent indigenous organizations and must include (1) primary health education with activities including conversations with the community, the development of books in their own language, and training for indigenous health workers, teachers, and community leaders; (2) recognition of the major risk factors by the community and the identification of the times of the year when these risks are increased; (3) implementation of control methods that reduce the possibility of human–vector contact, such as pyrethroid impregnated bednets, spraying the leaves of the palm before roofing houses, as have been implemented in other countries [48]; (4) teaching the communities how to handle the wild animals that are a part of their diet to avoid possible contamination in the event that these animals are infected with *T. cruzi*; (5) installation of a continuous entomological and serological monitoring program in these communities to be managed by indigenous health workers and supervised by competent authorities; and (6) treatment of the infected people.

## Conclusions

Active transmission of *T. cruzi* is present in four indigenous communities of the SNSM, Colombia with overlap between the domestic and sylvatic transmission cycles, which primarily results from the social, ecological, environmental and cultural conditions of the indigenous people.

## Competing interests

The authors declare that they have no competing interests.

## Authors’ contributions

Conceived and designed the experiments: AMMJ, LAAU, JCD, SO, AS, OTC. Performed the experiments: AMMJ, LAAU, JCD, SO. Analyzed the data: AMMJ, LAAU, JCD, SO, AS, OTC. Wrote the paper: AMMJ, AS, SO, OTC. All authors read and approved the final manuscript.
